# Response to Bakus et al. re: “Correlations Between Department and Training Program Online Presence and Women in Orthopedic Surgery Training”

**DOI:** 10.1089/whr.2024.0002

**Published:** 2024-02-15

**Authors:** Kimberly Templeton, Mary Zimmerman, Dorothy Hughes, Sarah Adkins

**Affiliations:** ^1^ Department of Orthopaedic Surgery, University of Kansas Medical Center, Kansas City, Kansas, USA.; ^2^ Department of Population Health, University of Kansas School of Medicine, Kansas City, Kansas, USA.; ^3^ Department of Population Health, University of Kansas, School of Medicine, Salina, Kansas, USA.; ^4^ University of Kansas School of Medicine, Kansas City, Kansas, USA.

We appreciate the editorial from Bakus et al.^1^ regarding our article, *Correlations between department and training program on-line presence and women in orthopaedic surgery training*^2^ and concur with their comments and recommendations. Improving diversity in orthopedic surgery requires an intentional, multifaceted approach, including recruiting and supporting women as academic faculty and then highlighting them and their accomplishments, including on department websites and social media accounts. Unfortunately, women faculty, especially in mid and late career, can become “invisible” and risk not having their accomplishments appropriately acknowledged and celebrated.^3^

As Bakus et al.^1^ note, medical students interested in orthopedic surgery cannot visit every program to assess culture and thus rely on either word-of-mouth or information provided by the programs. As we noted in our article, there has been significant increase in the use of social media by orthopedic departments and programs during the recent COVID-19 pandemic, and this can be an important tool in reaching students. However, just as important as having these accounts is the type of content being posted on them and included on department websites, the latter often being the first source of information that students seek.

Our article demonstrated the correlation between the diversity that departments demonstrate on their websites and the gender composition of their training programs. We agree with Bakus et al.^1^ that communications from orthopedic programs, via social media or otherwise, should provide information regarding the broad expanse of activities, from research to clinical care to social activities. However, as also noted by Bakus et al.,^1^ content needs to be carefully developed, with an eye to how the program wants to be viewed by others, especially students. This includes highlighting the roles and accomplishments of women to help demonstrate to potential applicants the opportunities for and support of women.

Increasing gender diversity in orthopedic surgery requires intentional effort: as has been demonstrated over the past 2 decades, having women as the majority of medical students does not mean that orthopedic workforce gender diversity will improve. Improving diversity is also a self-perpetuating cycle: recruit more women into residency programs, then recruit and support them as academic faculty and leaders, and then highlight their accomplishments to then recruit more women into orthopedic surgery residencies. Without these and other approaches, orthopedic surgery will remain as the least diverse specialty in medicine.
